# Supplementary figures and images for: Polyadic synapses introduce unique wiring architectures in T5 cells of Drosophila
Source: PLoS One. 2025 Oct 23;20(10):e0334925. doi: 10.1371/journal.pone.0334925 (PMC12548851; doi:10.1371/journal.pone.0334925)

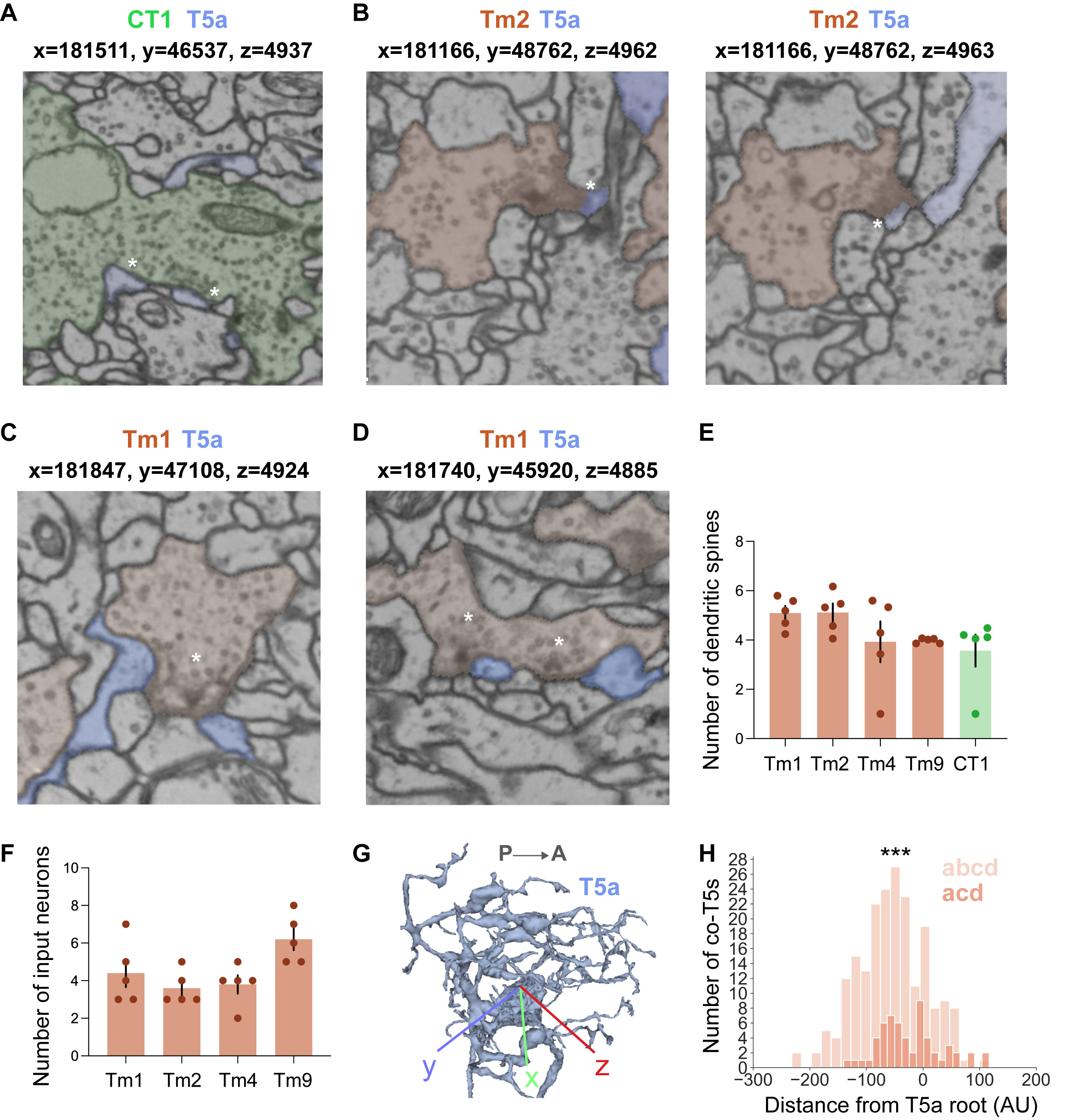

Supplement: S1 Fig — (A) Electron microscopy snapshot of one polyadic CT1-T5a synapse at a single z-plane (flywire.ai). Asterisks indicate the two T5a dendritic spines at the same CT1 presynaptic site. (B) Electron microscopy snapshot of one polyadic Tm2-T5a synapse at two sequential z-planes (flywire.ai). Asterisks indicate the two T5a dendritic spines at the same Tm2 presynaptic site. (C) Electron microscopy snapshot of one polyadic Tm1-T5a synapse at a single z-plane (flywire.ai). Asterisk indicates the three presynaptic T-bars. (D) Electron microscopy snapshot of two polyadic Tm1-T5a synapses at a single z-plane (flywire.ai). Asterisks indicate the two presynaptic T-bars. (E) Average number per neuronal input type of dendritic spines in Tm1-, Tm2-, Tm4-, Tm9- and CT1-to-T5a polyadic synapses of analysis. The normality of distribution was assessed with the use of Shapiro-Wilk test. Friedman test followed by Dunn’s post hoc test. Data is mean ± SEM. (F) Total number of input neurons per T5 dendrite of analysis. Data is mean ± SEM. (G) T5a posterior-anterior dendritic distribution in the three-dimensional embedding in LO1 (flywire.ai). (H) abcd- (light orange) and acd- (orange) derived co-T5 distribution from the root of T5a dendrites (nT5a=5) across Tm1- and Tm2-to-T5a synapses. The normality of distribution was assessed with the use of Shapiro-Wilk test. Two-tailed unpaired Student’s t-test, where ***p < 0.001. Detailed information is reported in S2 Table. (TIF) [file pone.0334925.s001.tif]

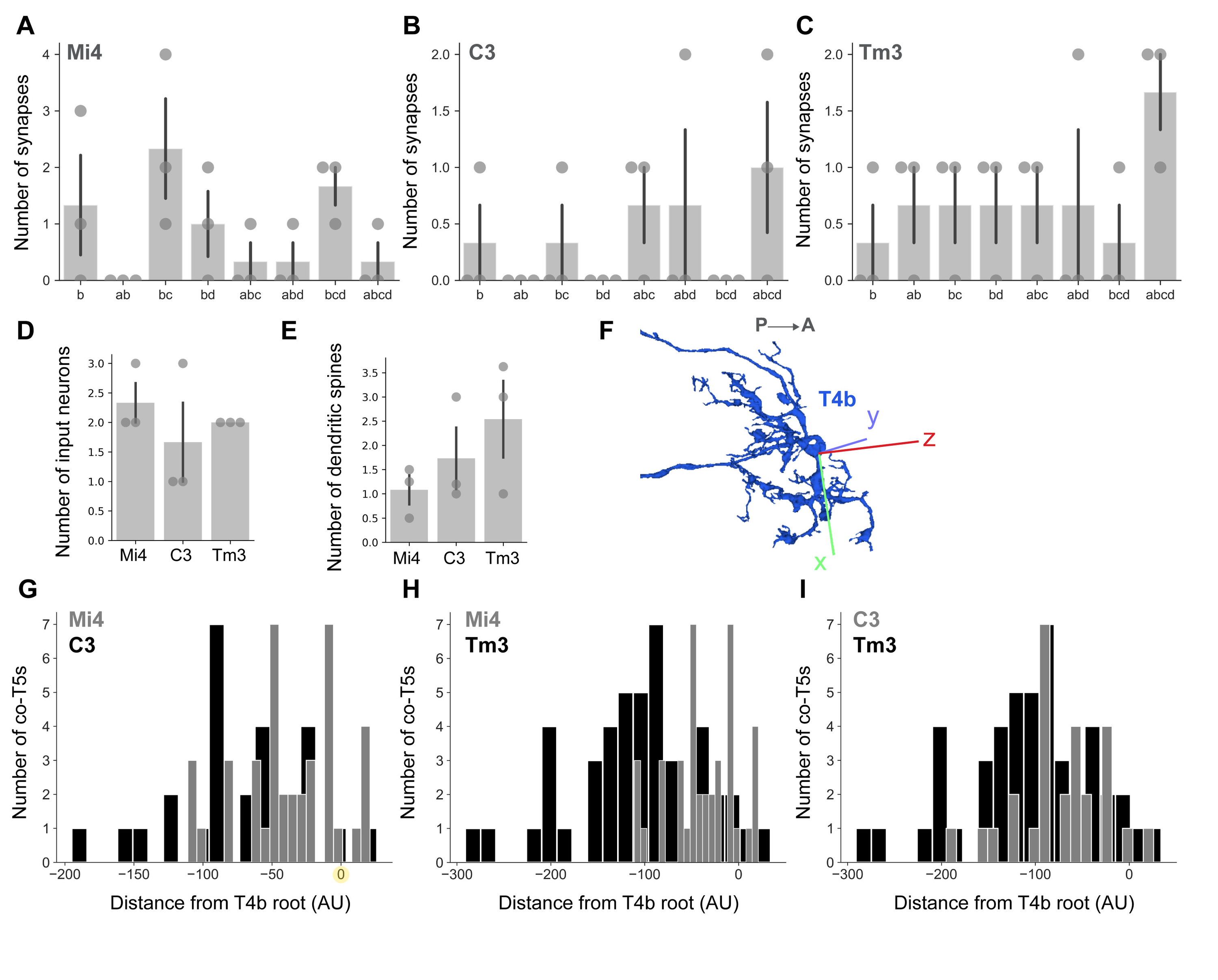

Supplement: S2 Fig — (A-C) Number of b, ab, bc, bd, abc, abd, bcd and abcd polyadic types in Mi4-, C3-, Tm3-to-T4b synapses (nT4b=3). Data is mean ± SEM. (D) Total number of input neurons per T4 dendrite of analysis. Data is mean ± SEM. (E) Average number per neuronal input type of dendritic spines in Mi4-, C3-, Tm3-to-T4b polyadic synapses of analysis. Data is mean ± SEM. (F) T4b posterior-anterior dendritic distribution in the three-dimensional embedding in medulla (flywire.ai, T4b ID 720575940615711338). (G-I) co-T5 distribution from the root of T4b dendrites (nT4b=3) across Mi4-, C3-, Tm3-to-T4b polyadic synapses. Dendritic root of T4b of analysis in yellow circle. The normality of distribution was assessed with the use of Shapiro-Wilk test. Two-tailed unpaired Student’s t-test, where *p < 0.05, **p < 0.01, ****p < 0.0001. Detailed information is reported in S2 Table. (TIF) [file pone.0334925.s002.tif]
