# Supplementary material for: Polyadic synapses introduce unique wiring architectures in T5 cells of Drosophila
Source: PLoS One. 2025 Oct 23;20(10):e0334925. doi: 10.1371/journal.pone.0334925 (PMC12548851; doi:10.1371/journal.pone.0334925)
Supplement: S1 Table — (DOCX) [file pone.0334925.s003.docx]

| **T5a** | 720575940643169933  **(Fig.1A,1C, S1A, S1C, S1D, S1G)** | 720575940625654759 | 720575940616396703  **(Fig.S1B)** | 720575940621106368 | 720575940626820538 |
| --- | --- | --- | --- | --- | --- |
| **Tm1** | 720575940620976493  (Fig. S1D)  720575940608883465 (Fig.1A, 1C, S1C)  720575940623583428  720575940627201308  720575940619723643  720575940640955088  720575940627479836 | 720575940622364961  720575940621146733  720575940608883465  720575940613587935  720575940616994134 | 720575940620523540  720575940608195339  720575940613587935  720575940634579135 | 720575940621146733  720575940633875117  720575940643191949 | 720575940616994134  720575940621146733  720575940613587935 |
| **Tm2** | 720575940630551670  720575940622364961 (Fig.1A)  720575940640230259  720575940621868276  720575940623081942 | 720575940632142904  720575940640453437  720575940640230259 | 720575940630308663  (Fig.S1B)  720575940640947152  720575940619708888  720575940632142904 | 720575940625164327  720575940615731359  720575940640453437 | 720575940619708888  720575940632142904  720575940615731359 |
| **Tm4** | 720575940637976666  (Fig.1A)  720575940620745163 | 720575940621582401  720575940620745163  720575940620782171  720575940637976666 | 720575940639445582  720575940620782171  720575940614824338  720575940627035198 | 720575940625442492  720575940621582401  720575940615720258  720575940613610642 | 720575940638140506  720575940620782171  720575940615720258  720575940639445582  720575940620745163 |
| **Tm9** | 720575940616384742  (Fig.1A)  720575940628740039  720575940613374873  720575940617906589  720575940620814356  720575940630425564  720575940624565296 | 720575940613374873  720575940620814356  720575940609254851  720575940626242348  720575940623771061 | 720575940610056654  720575940637539806  720575940626242348  720575940620680277  720575940631934039  720575940626578965  720575940653151649  720575940625797936 | 720575940626254892  720575940620814356  720575940623771061  720575940621776664  720575940626242348  720575940617906589 | 720575940625797936  720575940626242348  720575940623771061  720575940610056654  720575940620814356 |
| **CT1** | 720575940626979621  (Fig.1A, S1A) | 720575940626979621 | 720575940626979621 | 720575940626979621 | 720575940626979621 |
