# Supplementary material for: Polyadic synapses introduce unique wiring architectures in T5 cells of Drosophila
Source: PLoS One. 2025 Oct 23;20(10):e0334925. doi: 10.1371/journal.pone.0334925 (PMC12548851; doi:10.1371/journal.pone.0334925)
Supplement: S2 Table — (DOCX) [file pone.0334925.s004.docx]

| **Figure** | **Comparison** | **Statistical test** | **p-value** | **Significance** |
| --- | --- | --- | --- | --- |
| Figure 1E | Tm1-a, ab, ac, ad, abc, abd, acd, abcd | Friedman test followed by Dunn’s multiple comparisons test | p=0.0012 | ** |
| Figure 1F | Tm2-a, ab, ac, ad, abc, abd, acd, abcd | Friedman test followed by Dunn’s multiple comparisons test | p=0.0011 | ** |
| Figure 1G | Tm4-a, ab, ac, ad, abc, abd, acd, abcd | Friedman test followed by Dunn’s multiple comparisons test | p=0.0127 | * |
| Figure 1H | Tm9-a, ab, ac, ad, abc, abd, acd, abcd | Friedman test followed by Dunn’s multiple comparisons test | p=0.0005 | *** |
| Figure 1I | CT1-a, ab, ac, ad, abc, abd, acd, abcd | Friedman test followed by Dunn’s multiple comparisons test | p=0.0007 | *** |
| Figure 2B | Tm1_a(n=2) vs Tm2_a (n=2)  Tm1_a vs Tm4_a (n=1)  Tm1_a vs Tm9_a (n=5)  Tm1_a vs CT1_a (n=6)  Tm2_a vs Tm4_a  Tm2_a vs Tm9_a  Tm2_a vs CT1_a  Tm4_a vs Tm9_a  Tm4_a vs CT1_a  Tm9_a vs CT1_a | Kruskal-Wallis test followed by Mann–Whitney U post hoc test with Bonferroni correction | p=1.0000  p=1.0000  p=0.9524  p=0.9524  p=1.0000  p=1.0000  p=0.9524  p=1.0000  p=1.0000  p=0.0794 | ns |
| Figure 2C | Tm1_ab (n=1) vs Tm2_ab (n=2)  Tm1_ab vs Tm4_ab (n=3)  Tm1_ab vs Tm9_ab (n=7)  Tm1_ab vs CT1_ab (n=8)  Tm2_ab vs Tm4_ab  Tm2_ab vs Tm9_ab  Tm2_ab vs CT1_ab  Tm4_ab vs Tm9_ab  Tm4_ab vs CT1_ab  Tm9_ab vs CT1_ab | Kruskal-Wallis test followed by Mann–Whitney U post hoc test with Bonferroni correction | p=1.0000  p=1.0000  p=1.0000  p=1.0000  p=1.0000  p=1.0000  p=1.0000  p=1.0000  p=1.0000  p=0.1757 | ns |
| Figure 2D | Tm1_ac (n=2) vs Tm2_ac (n=3)  Tm1_ac vs Tm4_ac (n=3)  Tm1_ac vs Tm9_ac (n=4)  Tm1_ac vs CT1_ac (n=3)  Tm2_ac vs Tm4_ac  Tm2_ac vs Tm9_ac  Tm2_ac vs CT1_ac  Tm4_ac vs Tm9_ac  Tm4_ac vs CT1_ac  Tm9_ac vs CT1_ac | Kruskal-Wallis test followed by Mann–Whitney U post hoc test with Bonferroni correction | p=1.0000  p=1.0000  p=1.0000  p=1.0000  p=1.0000  p=1.0000  p=1.0000  p=1.0000  p=1.0000  p=0.5714 | ns |
| Figure 2E | Tm1_ad (n=0) vs Tm2_ad (n=2)  Tm1_ad vs Tm4_ad (n=5)  Tm1_ad vs Tm9_ad (n=41)  Tm1_ad vs CT1_ad (n=1)  Tm2_ad vs Tm4_ad  Tm2_ad vs Tm9_ad  Tm2_ad vs CT1_ad  Tm4_ad vs Tm9_ad  Tm4_ad vs CT1_ad  Tm9_ad vs CT1_ad | Kruskal-Wallis test followed by Mann–Whitney U post hoc test with Bonferroni correction | p=1.0000  p=1.0000  p=0.9888  p=1.0000  p=1.0000  p=1.0000  p=1.0000  p=0.0530  p=1.0000  p=0.9888 | ns |
| Figure 2F | Tm1_abc (n=11) vs Tm2_abc (n=9)  Tm1_abc vs Tm4_abc (n=7)  Tm1_abc vs Tm9_abc (n=3)  Tm1_abc vs CT1_abc (n=22)  Tm2_abc vs Tm4_abc  Tm2_abc vs Tm9_abc  Tm2_abc vs CT1_abc  Tm4_abc vs Tm9_abc  Tm4_abc vs CT1_abc  Tm9_abc vs CT1_abc | One-way ANOVA test followed by post-hoc pairwise t-tests with Bonferroni correction | p=1.0000  p=1.0000  p=0.4538  p=0.0276  p=1.0000  p=1.0000  p=0.0004  p=0.9476  p=0.0036  p=0.2233 | ns  ns  *  ns  ns  ***  ns  **  ns |
| Figure 2G | Tm1_abd (n=15) vs Tm2_abd (n=5)  Tm1_abd vs Tm4_abd (n=16)  Tm1_abd vs Tm9_abd (n=30)  Tm1_abd vs CT1_abd (n=17)  Tm2_abd vs Tm4_abd  Tm2_abd vs Tm9_abd  Tm2_abd vs CT1_abd  Tm4_abd vs Tm9_abd  Tm4_abd vs CT1_abd  Tm9_abd vs CT1_abd | Kruskal-Wallis test followed by Mann–Whitney U post hoc test with Bonferroni correction | p=1.0000  p=1.0000  p=0.0010  p=0.0001  p=1.0000  p=0.1164  p=0.0171  p=0.0796  p=0.0068  p=0.0000 | ns  ns  ***  ****  ns  ns  *  ns  **  **** |
| Figure 2H | Tm1_acd (n=10) vs Tm2_acd (n=15)  Tm1_acd vs Tm4_acd (n=13)  Tm1_acd vs Tm9_acd (n=15)  Tm1_acd vs CT1_acd (n=4)  Tm2_acd vs Tm4_acd  Tm2_acd vs Tm9_acd  Tm2_acd vs CT1_acd  Tm4_acd vs Tm9_acd  Tm4_acd vs CT1_acd  Tm9_acd vs CT1_acd | Kruskal-Wallis test followed by Mann–Whitney U post hoc test with Bonferroni correction | p=0.2461  p=0.0522  p=0.0014  p=0.5597  p=1.0000  p=0.0790  p=0.1234  p=1.0000  p=0.0386  p=0.0052 | ns  ns  **  ns  ns  ns  ns  ns  *  ** |
| Figure 2I | Tm1_abcd (n=53) vs Tm2_abcd (n=73)  Tm1_abcd vs Tm4_abcd (n=19)  Tm1_abcd vs Tm9_abcd (n=36)  Tm1_abcd vs CT1_abcd (n=33)  Tm2_abcd vs Tm4_abcd  Tm2_abcd vs Tm9_abcd  Tm2_abcd vs CT1_abcd  Tm4_abcd vs Tm9_abcd  Tm4_abcd vs CT1_abcd  Tm9_abcd vs CT1_abcd | One-way ANOVA test followed by post-hoc pairwise t-tests with Bonferroni correction | p=0.1890  p=1.0000  p=0.0000  p=0.0000  p=1.0000  p=0.0003  p=0.0000  p=0.0181  p=0.0001  p=0.0000 | ns  ns  ****  ****  ns  ***  ****  *  ****  **** |
| Figure 3C | Tm1-CT1 | Two-tailed unpaired Student’s t-test | p=0.000161 | *** |
| Figure 3D | Tm2-CT1 | Two-tailed unpaired Student’s t-test | p=0.000001 | **** |
| Figure 3E | Tm4-CT1 | Two-tailed unpaired Student’s t-test | p=0.000003 | **** |
| Figure 3F | Tm9-CT1 | Two-tailed unpaired Student’s t-test | p=0.000000 | **** |
| Figure S1H | abcd-acd | Two-tailed unpaired Student’s t-test | p=0.000291 | *** |
| Figure S2G | Mi4-C3 | Two-tailed unpaired Student’s t-test | p=0.001785 | ** |
| Figure S2H | Mi4-Tm3 | Two-tailed unpaired Student’s t-test | p=0.000000 | **** |
| Figure S2I | C3-Tm3 | Two-tailed unpaired Student’s t-test | p=0.021370 | * |
